# Virological and Clinical Determinants of the Magnitude of Humoral Responses to SARS-CoV-2 in Mild-Symptomatic Individuals

**DOI:** 10.3389/fimmu.2022.860215

**Published:** 2022-04-28

**Authors:** Edwards Pradenas, Maria Ubals, Víctor Urrea, Clara Suñer, Benjamin Trinité, Eva Riveira-Muñoz, Silvia Marfil, Carlos Ávila-Nieto, María Luisa Rodríguez de la Concepción, Ferran Tarrés-Freixas, Josep Laporte, Ester Ballana, Jorge Carrillo, Bonaventura Clotet, Oriol Mitjà, Julià Blanco

**Affiliations:** ^1^ IrsiCaixa AIDS Research Institute, Badalona, Spain; ^2^ Infectious Diseases Department, Fight against AIDS Foundation (FLS), Germans Trias i Pujol Hospital, Badalona, Spain; ^3^ Germans Trias i Pujol Research Institute (IGTP), Badalona, Spain; ^4^ CIBERINFEC, ISCIII, Madrid, Spain; ^5^ Chair in AIDS and Related Diseases, University of Vic–Central University of Catalonia (UVic-UCC), Vic, Spain

**Keywords:** COVID-19, seroconversion, neutralizing antibodies, viral load, humoral response, symptoms

## Abstract

**Background:**

Evidence on the determinants of the magnitude of humoral responses and neutralizing titers in individuals with mild COVID-19 is scarce.

**Methods:**

In this cohort study of mild COVID-19 patients, we assessed viral load (VL) by RT-qPCR at two/three time points during acute infection, and anti-SARS-CoV-2 antibodies by ELISA and plasma neutralizing responses using a pseudovirus assay at day 60.

**Results:**

Seventy-one individuals (65% female, median 42 years old) were recruited and grouped into high viral load (VL) >7.5 Log_10_ copies/mL (n=20), low, VL ≤7.5 Log_10_ copies/mL (n=22), or as Non-early seroconverters with a positive PCR (n=20), and healthy individuals with a negative PCR (n=9). Individuals with high or low VL showed similar titers of total neutralizing antibodies at day 60, irrespective of maximal VL or viral dynamics. Non-early seroconverters had lower antibody titers on day 60, albeit similar neutralizing activity as the groups with high or low VL. Longer symptom duration and older age were independently associated with increased humoral responses.

**Conclusions:**

In mild SARS-CoV-2-infected individuals, the duration of symptoms and age (but not VL) contribute to higher humoral responses.

## Introduction

Large efforts to understand the Coronavirus Disease 2019 (COVID-19) pathology suggest that infected patients elicit a rapid humoral response against the severe acute respiratory syndrome coronavirus 2 (SARS-CoV-2). Most patients seroconvert 19 days after symptom onset (1), though the kinetics of IgM and IgG antibodies is heterogeneous (2). Elicited antibodies show reactivity against multiple viral proteins, including the outer Spike (S) protein, which is the target of neutralizing antibodies (3). These include mainly, but not exclusively, antibodies blocking the binding of the S protein to the ACE-2 receptor through interaction with different epitopes of the receptor-binding domain (RBD) (4–10).

Although there is not a clear clinical definition of protective immunity in humans, neutralizing antibodies, which are elicited in most infected individuals, are able to protect golden Syrian hamsters from the acquisition of SARS-CoV-2 infection (9, 11) and are thought to play a relevant role in viral clearance after natural infection (12). Moreover, neutralizing antibodies generated by natural infection seem to be long-lasting (13) and correlate with protection against clinical reinfection (14, 15). Paradoxically, individuals with severe COVID-19 produce high-titers of antibodies (1, 16), while mild or asymptomatic infection leads to lower antibody titers or even lack of seroconversion (17).

Most of the knowledge generated on humoral responses against SARS-CoV-2 is based on severe/hospitalized patients. However, epidemiological data indicate that up to 80% of infected individuals present with mild disease (18). Importantly, there is an undetermined number of infected individuals, up to 40% in some studies, that do not develop symptoms (19). Overall, the heterogeneity of clinical trajectories observed after SARS-CoV-2 infection has been linked to different dynamics of immune responses, being an early innate and adaptive responses associated with early control of the infection and a mild clinical course, while late appearance of antibodies could be associated with more severe disease (20). It is, therefore, essential to gain a more comprehensive understanding of the antibody response to SARS-CoV-2 infection that captures the case-mix of disease pathways, particularly in patients with asymptomatic or mild COVID-19.

Here, we hypothesized that the degree of antigenic exposure could be a major determinant of the level of the humoral immune response. To evaluate this hypothesis, we designed the CIRCUS study and determined the titer of antibodies (total and neutralizing) in samples from individuals with well-defined viral load (VL) dynamics during acute infection, recruited from a previous randomized-controlled trial of COVID-19 cases and their contacts.

## Materials and Methods

### Study Design and Participants

This was an observational, prospective, and comparative pilot study: Characterizing the Immune Response to SARS-CoV-2 Under well-defined infection Settings, the CIRCUS study. The study aimed to characterize immune responses to SARS-CoV-2 among participants of the PEP CoV-2 “CQ4COV19” and Eudra CT 2020-001031-27 studies (21). Briefly, the PEP CoV-2 Study was a cluster-randomized clinical trial conducted during March and April 2020 in Catalonia (North-East Spain) to investigate the efficacy of hydroxychloroquine to treat and prevent COVID-19. The trial included two types of participants: mild confirmed cases of COVID-19 (“cases”) and asymptomatic adults who had a recent history of close-contact exposure to a PCR-confirmed COVID-19 case (“contacts”). Serial oral and nasopharyngeal swab samples were obtained on days 0, 3, and 7 for cases, and days 0 and 14 for contacts. The presence of SARS-CoV-2 was investigated from nasopharyngeal swabs, and viral load was quantified by RT-qPCR as described below. For contacts, IgM and IgG antibodies were detected from fingertip blood at day 14 visit using a rapid test (VivaDiag™ COVID-19 IgM/IgG) (22).

Participants of the CIRCUS study were selected among adult individuals (age ≥18 years) allocated in the control arm of the PEP CoV-2 trial and their close contacts; therefore, participants did not receive any investigational product. To characterize the impact of viral load on the magnitude of humoral responses to SARS-CoV-2, we defined four groups of patients from this cohort. The Non-early seroconverter group included 20 contacts with an acute SARS-CoV-2 infection characterized by a positive RT-qPCR test on days 0 and 14, and a negative result in the rapid antibody tests on day 14. The High VL group included 20 cases with an acute SARS-CoV-2 infection characterized by a positive RT-qPCR test, a maximum VL in nasopharyngeal swabs >7.5 Log_10_ copies/mL during acute infection (i.e., days 0, 3, or 7). The Low VL group included 22 cases with an acute SARS-CoV-2 infection characterized by a positive RT-qPCR test, a maximum VL in nasopharyngeal swabs <7.5 Log_10_ copies/mL during acute infection (days 0, 3, and 7). The control group consisted of 9 contacts without SARS-CoV-2 infection, confirmed by a negative RT-qPCR test (days 0 and 14), and a negative result in the rapid antibody tests (day 14). Cut-off VL value was selected according to virological data from the original PEP CoV-2 study (21).

A follow-up visit was scheduled 60 days after symptom onset or diagnosis by PCR ( ± 7 days). A blood sample and a nasopharyngeal swab were collected. Participants were interviewed about the presence of specific comorbidities and risk factors (smokers, hypertension, dyslipidemia, obesity, diabetes mellitus, respiratory or autoimmune disease). Respiratory diseases included Chronic Obstructive Pulmonary Disease (COPD) and asthma. Obesity was defined as a Body Mass Index (BMI)>30. Epidemiological and clinical data were obtained from CQ4COV19 clinical trial (age, gender, time from diagnosed infection, treatment, severity of infection, peak VL, VL follow-up, cumulative antigen exposure and time to symptom resolution). In this trial, resolution of symptoms was assessed sequentially using a symptoms questionnaire designed to gather information on the type of symptom and last day experienced; complete resolution was considered when no COVID-19–related symptoms were reported at all. Participants received telephonic interviews on days 3, 7, 14, and 28. The following symptoms were considered as COVID-19-related: dyspnea, fever, cough, sudden olfactory or gustatory loss, rhinitis, headache, thoracic pain (21). Severity of SARS-CoV-2 infection was assessed using the WHO scale (23).

### SARS-CoV-2 PCR Detection and Viral Load Quantification

RNA extraction was performed using the Viral RNA/Pathogen Nucleic Acid Isolation kit (Thermo Fisher), optimized for a KingFisher instrument (Thermo Fisher), following manufacturer’s instructions. PCR amplification was based on the 2019-Novel Coronavirus Real-Time RT-PCR Diagnostic Panel guidelines and protocol developed by the American Center for Disease Control and Prevention (24). Briefly, a 20 μL PCR reaction was set up containing 5 μL of RNA, 1.5 μL of N2 or RNAseP primers and probe (2019-nCov CDC EUA Kit, Integrated DNA Technologies) and 10 μL of GoTaq 1-Step RT-qPCR (Promega). Thermal cycling was performed at 50°C for 15 min for reverse transcription, followed by 95°C for 2 min and then 45 cycles of 95°C for 10 sec, 56°C for 15 sec and 72°C for 30 sec in the Applied Biosystems 7500 or QuantStudio5 Real-Time PCR instruments (Thermo Fisher). For absolute quantification, a standard curve was built using 1/5 serial dilutions of a SARS-CoV-2 plasmid (2019-nCoV_N_Positive Control, 200 copies/μL, Integrated DNA Technologies) and run in parallel in all PCR determinations. The VL of each sample was determined in triplicate, and mean VL (in copies/mL) was extrapolated from the standard curve and corrected by the corresponding dilution factor. RNAseP gene amplification was performed in duplicate for each sample as an amplification control.

### Humoral Response Determination

The humoral response against SARS-CoV-2 was evaluated with an in-house sandwich- ELISA using the following antigens (Sino Biological): S1+S2 protein, RBD (Arg319-Phe541), both potentially contributing to neutralizing activity; and whole nucleocapsid protein (NP), which is unrelated to neutralizing capacity. Nunc MaxiSorp plates were coated with 50 μL of anti-6x-His antibody clone HIS.H8 (2 μg/mL, Thermo Fisher) in PBS overnight at 4°C. After washing, plates were blocked with 1% BSA (Miltenyi Biotec in PBS) for two hours at room temperature. Antigens were added at 1 μg/mL (50 μL/well) and incubated overnight at 4°C. Plasma samples were heat-inactivated before use (56°C for 30 min) and analyzed in duplicate in antigen-coated and antigen-free wells in the same plate. Serial dilutions of a positive plasma sample were used as standard. A pool of pre-pandemic plasmas from healthy controls was used as a negative control. Standards, negative control, and plasma samples were diluted in blocking buffer and were incubated (50 μL/well) for one hour at room temperature. The HRP-conjugated F(ab’)2-goat anti-human IgG (Fc specific, Jackson ImmunoResearch) was then incubated for 30 minutes at room temperature. Plates were revealed with o-Phenylenediamine dihydrochloride (Sigma-Aldrich), and the reaction was stopped using 4N of H_2_SO_4_ (Sigma-Aldrich). Optical density (OD) at 492 nm with noise correction at 620 nm were used to calculate specific signals for each antigen after subtracting the antigen-free well signal for each sample. Standard curves were fitted to a 5-parameter logistic curve, and data was quantitatively expressed as arbitrary units (AU) according to the standard.

### Pseudovirus Neutralization Assay

HIV reporter pseudoviruses expressing SARS-CoV-2 S protein and Luciferase were generated. pNL4-3.Luc.R-.E- was obtained from the NIH AIDS Reagent Program (25). SARS-CoV-2.SctΔ19 was generated (GeneArt) from the full protein sequence of SARS-CoV-2 spike with a deletion of the last 19 amino acids in C-terminal (26), human-codon optimized and inserted into pcDNA3.4-TOPO. Expi293F cells were transfected using ExpiFectamine293 Reagent (Thermo Fisher) with pNL4-3.Luc.R-.E- and SARS-CoV-2.SctΔ19 at an 8:1 ratio, respectively. Control pseudoviruses were obtained by replacing the S protein expression plasmid with a VSV-G protein expression plasmid as reported (27). Supernatants were harvested 48 h after transfection, filtered at 0.45 μm, frozen, and titrated on HEK293T cells overexpressing WT human ACE-2 (Integral Molecular). This neutralization assay has been previously validated in a larger subset of samples (1).

Neutralization assays were performed in duplicate. Briefly, in Nunc 96-well cell culture plates (Thermo Fisher), 200 TCID50 of pseudovirus were preincubated with three-fold serial dilutions (1/60–1/14,580) of heat-inactivated plasma samples for 1 h at 37°C. Then, 2x10^4^ HEK293T/hACE2 cells treated with DEAE-Dextran (Sigma-Aldrich) were added. Results were read after 48 hours using the EnSight Multimode Plate Reader and BriteLite Plus Luciferase reagent (PerkinElmer). The values were normalized and the inhibitory dilution (ID) 50 (the reciprocal dilution inhibiting 50% of the infection) was calculated by plotting and fitting the log of plasma dilution versus response to a 4-parameters equation in Prism 8.4.3 (GraphPad Software).

### Statistical Analysis

Continuous variables were described using medians and the interquartile range (IQR, defined by the 25^th^ and 75^th^ percentiles) or the mean and the standard error of the mean (SEM), whereas categorical variables were reported as percentages over available data. The different groups were compared using the nonparametric Mann-Whitney and Kruskal-Wallis with Dunn’s multiple comparison tests. Correlations were assessed by Spearman test. Multivariate linear regression analyses were performed to assess independent associations of gender, age, symptoms duration and VL with the measured humoral responses at day 60 (Log_10_ transformed). Two-sided p-value ≤0.05 was considered statistically significant. All analyses were performed with GraphPad Prism 8.4.3 (GraphPad Software, Inc.) and R version 4.0 (R Foundation for Statistical Computing).

## Results

### Study Cohort

The CIRCUS study enrolled 71 participants who had been exposed to a COVID-19 case no more than 5 days before enrollment and were asymptomatic or had mild symptoms with onset up to 3 days before enrollment. Main characteristics are described in **Table 1**. The median [IQR] age of individuals was 43 [30-52] years, and 64.8% (46/71) were female. Most of the individuals were either healthcare workers (60.6%) or nursing home workers (14.1%); the remaining were household contacts (22.5%). The main comorbidities were obesity, respiratory disease, dyslipidemia, and hypertension.

**Table 1:** Description of participants.

	Non-early seroconverter	High VL	Low VL	Control
	n = 20	n = 20	n = 22	n = 9
Gender (female), n (%)	11 (55)	12 (60)	18 (82)	5 (56)
Age (years), median [IQR]	45 [36-54]	37 [27-51]	40 [30-54]	47 [32-52]
**Type of contact with index case, n (%)**				
Household contact	9 (45)	1 (5)	1 (5)	5 (56)
Healthcare worker	4 (20)	18 (90)	17 (77)	4 (44)
Nursing home worker	7 (35)	0 (0)	3 (14)	0 (0)
Unknown	0 (0)	1 (5)	1 (5)	0 (0)
**Coexisting disease and other factors, n (%)**				
None	8 (40)	15 (75)	12 (54)	1 (11)
Smoker	5 (25)	1 (5)	1 (5)	5 (56)
HT or Dyslipidemia	4 (20)	0 (0)	5 (22)	1 (11)
Obesity or DM	3 (15)	0 (0)	5 (22)	3 (33)
Respiratory disease	3 (15)	4 (20)	1 (5)	1 (11)
Autoimmune disease	0 (0)	0 (0)	1 (5)	0 (0)
**Clinical presentation**				
Symptoms (yes), n (%)	5 (25)	19 (95)	19 (86)	—

IQR, interquartile range (25th and 75th percentiles).

For our analysis on determinant factors of antibody response, RT-qPCR positive individuals were categorized into 3 groups: 20 individuals (28.2%) with maximum viral load >7.5 log_10_ RNA copies/mL (High VL); 22 individuals (31.0%) with maximum viral load <7.5 log_10_ RNA copies/mL (Low VL), and 20 individuals (28.2%) with a negative rapid antibody test on day 14 after a positive PCR result (Non-early seroconverter). A group of 9 RT-qPCR negative individuals was also included as control. **Table 1** summarizes the main demographical and clinical characteristics of individuals allocated in each of the immune response groups.

When we looked at the clinical presentation in each of the three infected groups, the proportion of symptomatic disease was significantly higher in the High VL group (95%) and Low VL group (86%) compared to Non-early seroconverter individuals (25%, chi-square test *p* < 0.001, **Table 1**). No significant differences were observed between the High and Low VL groups.

### Viral Dynamics and Seroconversion

**Figure 1:**
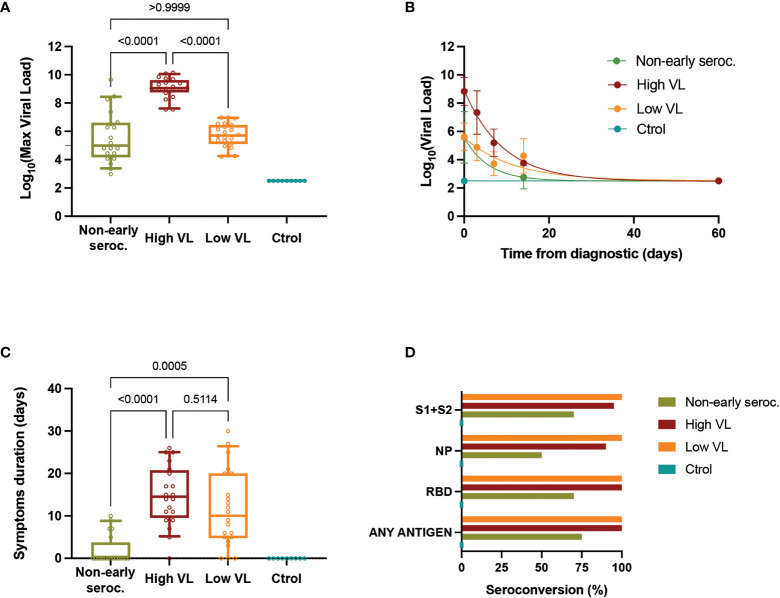
Viral load determinations. **(A)** Maximal values of VL for each individual. Boxes show the median and the 25^th^-75^th^ interquartile range and bars the 10^th^-90^th^ interquartile range. P values correspond to Kruskal-Wallis test with Dunn’s multiple comparisons. **(B)** VL dynamics in each group (mean ± SEM) with the best fit curve (single exponential decay). **(C)** Self-reported symptom duration for each individual. Boxes show the median and the 25^th^-75^th^ interquartile range and bars the 10^th^-90^th^ interquartile range. P values correspond to Kruskal-Wallis test with Dunn’s multiple comparisons. **(D)** Seroconversion (frequency of positive samples for the indicated individual antigens or for any of them) in the different groups assessed by ELISA at day 60 of follow-up.

The characterization of the study cohort included a virological and serological follow-up. The VL peak of Non-early seroconverter individuals was similar to that of the Low VL group and significantly lower than the High VL group (by definition >7.5 Log_10_ copies/mL, **Figure 1A**). The VL declined rapidly in the High VL group and slower in the Low VL group; Non-early seroconverter individuals showed a fast decay in VL, with only two positive samples 14 days after diagnosis (**Figure 1B**). The VL was associated with self-reported symptom duration, which was significantly lower in the Non-early seroconverter group and showed no significant differences between the Low and High VL groups (**Figure 1C**).

All individuals were tested 60 days after diagnosis to evaluate IgG humoral response by in-house ELISAs against the spike (S1+S2 protein), the RBD, and the NP. Of the 62 subjects with positive RT-qPCR for SARS-CoV-2, 49 (79.0%) had detectable IgG titers against all three antigens tested, while 5 (8.1%) individuals had no IgG antibodies against any antigen (all of them belonged to the Non-early seroconverter group). Antibodies against S1+S2 proteins and RBD were more frequently positive (89% and 90% of cases, respectively) than anti-NP antibodies (79%). The overall positivity (i.e., the proportion of individuals testing positive in at least one antigen) at day 60 was 100% for both the Low and High VL groups. The Non-early seroconverter group had a lower proportion of positive individuals to antibodies against S1+S2 proteins, RBD, and anti-NP antibodies: 70%, 70%, and 50%, respectively. The overall positivity (75%) was also lower in this group. All uninfected individuals had undetectable IgG antibodies (**Figure 1D**).

### Levels of Humoral Immunity and Neutralizing Activity

**Figure 2:**
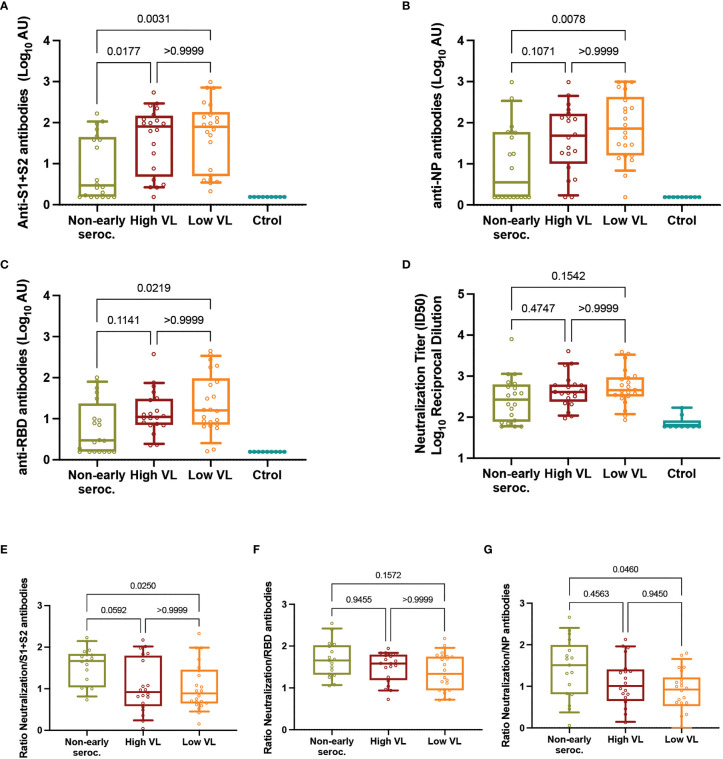
Neutralizing activity. Individual titers of **(A)** anti-S1+S2 antibodies, **(B)** anti-NP antibodies, **(C)** anti-RBD antibodies, and **(D)** neutralization. Individual ratios of neutralization titers to **(E)** anti-S1+S2, **(F)** anti-RBD, and **(G)** anti-NP antibodies. In all panels, boxes show the median and the 25^th^-75^th^ interquartile range and bars the 10^th^-90^th^ interquartile range. *P* values correspond to Kruskal-Wallis test with Dunn’s multiple comparisons. ID, inhibitory dilution; NP, nucleocapsid protein; VL, viral load.

The analysis of humoral responses at day 60 showed no differences between High and Low VL groups for anti-S1+S2, anti-NP, or anti-RBD responses. When comparing these two groups together with the Non-early seroconverter group, we observed significant differences with lower median IgG titer against all antigens (*p* < 0.05, **Figures 2A–C**). Using a neutralization assay with HIV-based pseudoviruses exposing the SARS-CoV-2 S or the VSV-G proteins, we analyzed all plasma samples using serial dilutions starting at 1/60 dilution (limit of detection). Specific neutralizing activity against SARS-CoV-2 was detected in 95% of RT-qPCR positive cases, including 85% in the Non-early seroconverter group. High and Low VL groups showed similar median values of neutralization titers, and the Non-early seroconverter group did not have significantly lower values compared to the other groups (**Figure 2D**).

Similar results were observed in an additional analysis in which Non-early seroconverters with High or Low VL (n=3 and n=17, respectively) were reclassified in the High and Low VL groups. These larger (n=23 High VL, n=39 Low VL) groups did not show differences in the levels of anti-S1+S2, anti-NP, anti-RBD or neutralizing antibodies at day 60 of follow-up (**Supplementary Figure 1**).

The unexpected high neutralization observed in Non-early seroconverter individuals, despite significantly lower antibody titers, suggested a good quality of neutralization in this group. To assess this possibility, we calculated the ratio of neutralizing titers and total antibodies for each individual as a proxy of antibody quality. The comparison of these ratios among groups showed significant differences for the ratio neutralization/anti-S1+S2 antibodies and anti-NP antibodies, being in both cases higher in Non-early seroconverter individuals (**Figures 2E, F**). In contrast, no impact of levels of anti-RBD antibodies on neutralization activity was observed (ratios similar among groups, **Figure 2G**).

### Analysis of Determinants of Humoral Response

**Figure 3:**
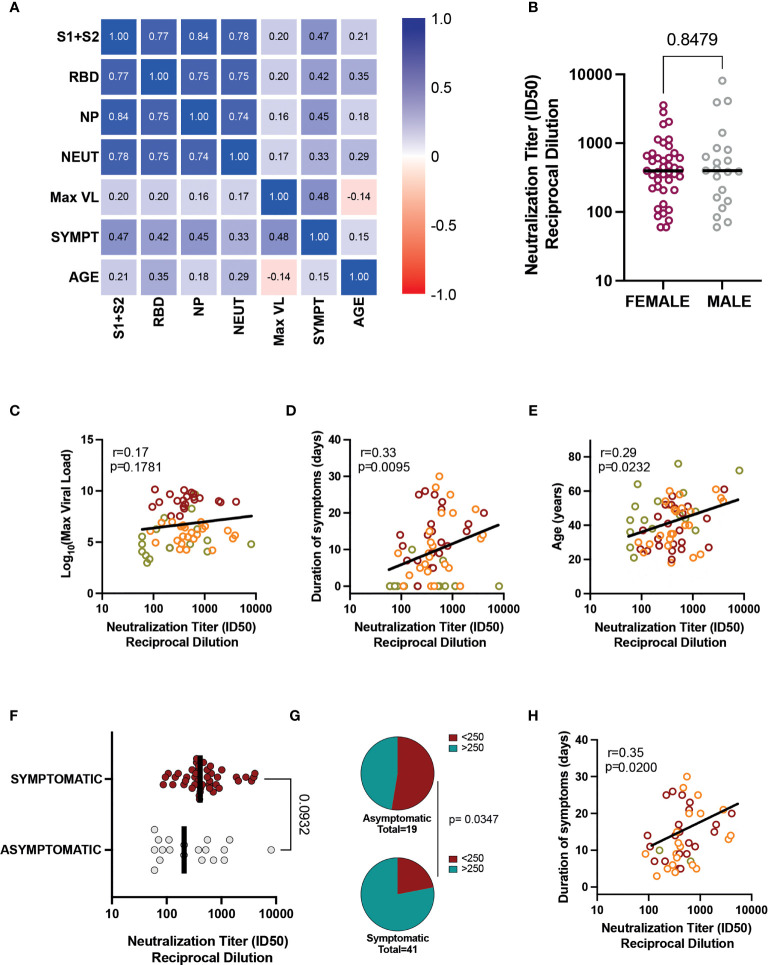
Associations between humoral responses and clinical/demographic characteristics. **(A)** Heatmap of correlations between total IgG, neutralizing antibody titer, VL, symptom duration, and age for all patients. **(B)** Neutralization titers in female and male participants (all groups). Bar indicates median values, and the *p*-value is shown (Mann-Whitney test). Correlation of neutralization titers with **(C)** VL, **(D)** Duration of Symptoms, and **(E)** Age. Individual values are color-coded: green for Non-early seroconverter individuals at diagnosis, orange for individuals with low viral load, and red for individuals with high viral load. Lines indicate linear regression of all values for illustrative purposes. The correlation coefficient and *p*-value (Spearman correlation test) are shown. **(F)** The impact of asymptomatic infection was assessed by comparing neutralizing titers between all asymptomatic (grey dots) and all symptomatic patients (red symbols). **(G)** Frequency of low and high neutralizers (cutoff value 1/250); the Fisher exact test *p*-value is shown. **(H)** Correlation between symptom duration and neutralization titer in symptomatic individuals. The correlation coefficient and *p*-value (Spearman correlation test) is shown. ID, inhibitory dilution; NP, nucleocapsid protein; RBD, receptor-binding domain; VL, viral load.

The multiple correlation analysis showed a positive association between the neutralizing antibody titer and the total IgG antibody levels against all antigens, with the highest association observed in anti-spike antibodies (r=0.78, **Figure 3A**). The results were similar when we performed the analysis for each group of patients separately (data not shown). In the analysis of determinants of humoral response, we adjusted for virological and demographical factors that could be associated with the presence of IgG antibodies and neutralizing activity. Gender was analyzed separately, showing no impact on neutralizing antibodies (**Figure 3B**). The maximum VL was not associated with neutralizing capacity (*p* = 0.17, **Figure 3C**), nor with IgG antibody titers against any antigen (anti-S1+S2: *p* = 0.11; anti-RBD: *p* = 0.12; anti-NP: *p* = 0.21, data not shown). In contrast, we observed that both the duration of symptoms and age were associated with the level of the humoral response. The duration of symptoms was positively associated with antibody titers (**Figure 3A**) and neutralizing activity (r = 0.33; *p* = 0.0095, **Figure 3D**), although the analysis by groups revealed a lack of correlation for the Non-early seroconverter group (r = 0.99; *p* = 0.72, data not shown), probably due to the higher frequency of asymptomatic individuals.

Older age correlated with increasing neutralizing antibody titer (r = 0.29; *p* = 0.023) (**Figure 3E**), but the correlation was not significant for the Non-early seroconverter group (r = 0.35; *p* = 0.14, data not shown). This correlation was also observed for anti-RBD antibodies (r = 0.35; *p* = 0.005), but not for anti-S1+S2 (r = 0.21; *p* = 0.10) and anti-NP (r = 0.18; *p* = 0.16) antibodies (**Figure 3A**).

We also performed multivariate linear regression analysis to identify the relationship of gender, age, duration of symptoms and VL with humoral responses. As summarized in **Supplementary Table 1**, duration of symptoms was independently associated with anti-S1+S2 and anti-NP antibody titers (*p* = 0.0018 and 0.0036, respectively). Both duration of symptoms and age were associated with anti-RBD antibody titers (*p* = 0.0062 and 0.0140, respectively), while exclusively age was significantly associated with neutralization titers (*p* = 0.0114, **Supplementary Table 1**). In summary, these analyses confirmed that duration of symptoms and age are the main determinants of the magnitude of humoral responses in our cohort.

To further assess the impact of asymptomatic infection on neutralization titers, we grouped patients into asymptomatic (n=19) or symptomatic (n=43), irrespective of the initial classification. Although neutralization titers tended to be lower in asymptomatic individuals, the comparison did not reach statistical significance due to the presence of high-neutralizers in the asymptomatic group (**Figure 3F**). Nevertheless, the frequency of low neutralizers (using a cutoff value of 1/250) (14, 16) was significantly lower in the asymptomatic group (**Figure 3G**), suggesting that asymptomatic individuals may interfere in the global analysis of the correlation of symptom duration and neutralizing responses. To avoid this potential artifact, we reanalyzed the data using only symptomatic individuals, which maintained a significant correlation (r = 0.35; *p* = 0.0200) between neutralization titers and symptom duration (**Figure 3H**), thus confirming the robust link between both parameters. Humoral responses (neutralizing or total antibodies) were not significantly influenced by other clinical or demographic characteristics such as gender, smoking, cardiovascular disease, obesity, respiratory disease, influenza vaccination, or residual symptoms (data not shown).

## Discussion

In this study of factors influencing the levels of neutralizing antibodies in asymptomatic and mildly symptomatic SARS-CoV-2-infected individuals, we observed that more than 90% of participants had detectable IgG antibodies 60 days after diagnosis. The proportion of serum antibody positivity was 100% in symptomatic individuals; however, it was lower in asymptomatic individuals, also leading to lower titers of neutralizing antibodies. This finding has already been described (1, 28, 29) and points to a relevant role of other arms of the immune system (innate immunity or cellular responses) in the early and effective control of SARS-CoV-2 infection, in the absence of detectable serum antibodies (30, 31).

Several studies have shown that humoral response is related to COVID-19 severity (1, 2, 32), which in turn is associated with age and gender (33) and has also been linked to VL (34). However, these reports analyze both hospitalized individuals and outpatients and are usually not designed to analyze these groups independently. Our study, which specifically focuses on outpatients to uncover the potential determinants of the magnitude of humoral responses, found that only age and symptom duration had a significant association. While age seems to correlate with neutralization and RBD antibodies, symptom duration showed a more consistent correlation with all humoral response parameters analyzed (anti-S1+S2, anti-RBD, anti-NP, and neutralizing antibodies). The correlation was still strong when looking at symptomatic individuals alone to avoid a potential confounding effect of asymptomatic individuals.

Finally, we did not observe an association between the elicitation of humoral responses and the different comorbidities; this should be interpreted with caution because the number of individuals with these characteristics did not allow us to perform a formal statistical analysis. Additionally, some comorbidities, such as hypertension, cardiovascular and respiratory diseases, have been found to be related to the COVID-19 severity (35); nevertheless, our study included only asymptomatic or mild symptomatic cases, explaining the fact that no differences were observed between groups with and without underlying diseases.

Our data is consistent with the notion that the early control of SARS-CoV-2 replication may be determinant in humoral responses. An effective control of infection by strong innate mechanisms or preexisting cross-reactive CTLs may limit the extent of SARS-CoV-2 replication (36, 37), and hence antigen levels and subsequent antibody development. In contrast, the failure to control viral replication may lead to sustained B cell activation and antibody generation, resulting in increased titers of humoral responses. The determinants of this early control are still unclear and involve the efficacy of both innate and adaptive responses (20). Cross-reactivity of SARS-CoV-2 and other common cold human coronaviruses have been reported not only for cellular responses but also for antibodies (38–40), mainly those directed against the S2 subunit of Spike glycoprotein (41). In our cohort, we could not analyze preexisting responses but we found surprisingly high levels of neutralizing antibodies in individuals that were seronegative 14 days after diagnosis. When examining the reasons behind this high activity, we observed that it seems to be related to a high quality of neutralizing antibodies against the S1 and S2 proteins, but not against the RBD. Whether this observation reflects the rapid expansion of preexisting memory B cell responses remains to be defined.

Our analysis is mainly limited by the reduced sample size, which is insufficiently powered to assess comorbidities as factors that could determine the humoral response. In addition, our findings are not necessarily transferable to newer SARS-CoV-2 variants that exhibit modified tissue tropism, transmissibility and potentially immune response pattern (42). These and other parameters should be investigated further, and larger studies with longer follow-ups will be needed to address this issue properly. In contrast, despite limited sample size, our data on VL dynamics clearly rule out an impact of the level of viral exposure (as determined in nasopharyngeal swabs) on humoral responses. Rather, we identified age and symptom duration, which showed a partial correlation with VL, as the main parameter determining humoral responses in mild COVID-19 patients.

## Data Availability Statement

The raw data supporting the conclusions of this article will be made available by the authors, without undue reservation.

## Ethics Statement

The studies involving human participants were reviewed and approved by Comité Ético Hospital Germans Trias i Pujol. The patients/participants provided their written informed consent to participate in this study.

## Author Contributions

JB, OM and BC designed and coordinated the study. EP, BT, SM, CA-N, MLR, FT-F and JC, performed and analyzed the neutralization and ELISA assays. ER-M and EB, performed viral load quantification. VU performed the statistical analysis. MU, CS and JL selected the patients and coordinated the data. EP, OM and JB drafted the manuscript, and all authors made substantial contributions to the revision of the subsequent versions. All authors approved the submitted version of the manuscript and agreed both to be personally accountable for their own contributions and to answer the questions related to the accuracy or integrity of any part of the work.

## Funding

This work was partially funded by Grifols, the *Departament de Salut* of the *Generalitat de Catalunya* (grant DSL016 to JB and Grant DSL015 to JC), the Spanish Health Institute Carlos III (Grant PI17/01518 and PI20/00093 to JB and PI18/01332 to JC), CERCA Programme/*Generalitat de Catalunya* 2017 SGR 252, and the crowdfunding initiatives #joemcorono, BonPreu/Esclat and Correos and Foundation Dormeur for the acquisition of the QuantStudio-5 real time PCR system. EP was supported by a doctoral grant from National Agency for Research and Development of Chile (ANID): Grant 72180406. CA-N was supported by a predoctoral grant from *Secretaria d’Universitats de la Generalitat de Catalunya* and the European Social Funds (2020-FI_B_00742). The funders had no role in study design, data collection and analysis, the decision to publish, or the preparation of the manuscript.

## Conflict of Interest

Outside the submitted work JB and JC are founders and shareholders of AlbaJuna Therapeutics, S.L. BC is founder and shareholder of AlbaJuna Therapeutics, S.L and AELIX Therapeutics, S.L.

The remaining authors declare that the research was conducted in the absence of any commercial or financial relationships that could be construed as a potential conflict of interest.

## Publisher’s Note

All claims expressed in this article are solely those of the authors and do not necessarily represent those of their affiliated organizations, or those of the publisher, the editors and the reviewers. Any product that may be evaluated in this article, or claim that may be made by its manufacturer, is not guaranteed or endorsed by the publisher.
