# Supplementary material for: Virological and Clinical Determinants of the Magnitude of Humoral Responses to SARS-CoV-2 in Mild-Symptomatic Individuals
Source: Front Immunol. 2022 Apr 28;13:860215. doi: 10.3389/fimmu.2022.860215 (PMC9097229; doi:10.3389/fimmu.2022.860215)
Supplement: Supplementary file 1 [file DataSheet_1.docx]

Supplementary Material

**Supplementary Figure 1.** **Merged analysis of all participants.** Participants were analyzed according to their maximal VL. Three Non-early seroconverter individuals were included in the High VL group (n=23) and 17 were included in the Low VL group (n=39). Control uninfected individuals were included for reference (n=9). The indicated quantitative parameters of humoral responses were analyzed at day 60. No statistical differences were observed (Mann-Whitney test).

|  |  |  |  |  |
| --- | --- | --- | --- | --- |
| **Neutralization** |  |  |  |  |
|  | Estimate | Std. Error | *p*-value |  |
| Gender (men) | 0.050094 | 0.129006 | 0.6992 |  |
| Age (year) | 0.01165 | 0.004456 | 0.0114 | * |
| Duration of symptoms (days) | 0.013253 | 0.007762 | 0.0932 |  |
| Viral Load (Log10 copies/mL) | 0.023485 | 0.033324 | 0.4838 |  |
|  |  |  |  |  |
| **anti-S1+S2 antibody titer** |  |  |  |  |
|  | Estimate | Std. Error | *p*-value |  |
| Gender (men) | -0.052055 | 0.224112 | 0.81716 |  |
| Age (year) | 0.010559 | 0.007742 | 0.17798 |  |
| Duration of symptoms (days) | 0.044127 | 0.013484 | 0.00181 | ** |
| Viral Load (Log10 copies/mL) | 0.015272 | 0.057891 | 0.79288 |  |
|  |  |  |  |  |
| **anti-NP antibody titer** |  |  |  |  |
|  | Estimate | Std. Error | *p*-value |  |
| Gender (men) | -0.039961 | 0.244766 | 0.8709 |  |
| Age (year) | 0.006731 | 0.008455 | 0.4293 |  |
| Duration of symptoms (days) | 0.044728 | 0.014726 | 0.0036 | ** |
| Viral Load (Log10 copies/mL) | 0.00127 | 0.063226 | 0.984 |  |
|  |  |  |  |  |
| **anti-RBD antibody titer** |  |  |  |  |
|  | Estimate | Std. Error | *p*-value |  |
| Gender (men) | 0.113802 | 0.173774 | 0.51517 |  |
| Age (year) | 0.015217 | 0.006003 | 0.01402 | * |
| Duration of symptoms (days) | 0.029688 | 0.010455 | 0.00625 | ** |
| Viral Load (Log10 copies/mL) | 0.001334 | 0.044888 | 0.97639 |  |
|  |  |  |  |  |

**Supplementary Table 1.** **Analysis of factors associated with humoral responses.** A multivariate linear regression analysis was performed to identify independent association of gender, age, duration of symptoms and VL with Neutralization or the indicated antibody titers. Asterisks denote significant association (*<0.05, **<0.001).
